# Evidence-Based Interventions that Promote Resident Wellness from the Council of Emergency Residency Directors

**DOI:** 10.5811/westjem.2019.11.42961

**Published:** 2020-02-21

**Authors:** Melissa Parsons, John Bailitz, Arlene S. Chung, Alexandra Mannix, Nicole Battaglioli, Michelle Clinton, Michael Gottlieb

**Affiliations:** * University of Florida College of Medicine, Department of Emergency Medicine, Jacksonville, Florida; † Northwestern University Feinberg School of Medicine, Department of Emergency Medicine, Chicago, Illinois; ‡ Maimonides Medical Center, Department of Emergency Medicine, Brooklyn, New York; ¶ Carilion Clinic, Department of Emergency Medicine, Roanoke, Virginia; || Rush Medical Center, Department of Emergency Medicine, Chicago, Illinois

## Abstract

Initiatives for addressing resident wellness are a recent requirement of the Accreditation Council for Graduate Medical Education in response to high rates of resident burnout nationally. We review the literature on wellness and burnout in residency education with a focus on assessment, individual-level interventions, and systemic or organizational interventions.

## BACKGROUND

Burnout syndrome was defined in the 1970s as a triad of emotional exhaustion (EE), depersonalization (DP), and a low sense of personal accomplishment (PA).^1^ Almost half of physicians report burnout, and emergency physicians (EP) are near the top of the list.^2^ In 2018, 48% of EPs reported burnout.^3^ Physician burnout has been shown to negatively correlate with patient safety, quality of care, physician professionalism, and patient satisfaction.^4^,^5^ In EPs, burnout was associated with increased frequency of self-reported, suboptimal patient care, including admitting or discharging patients early, not communicating effectively with patients, ordering more tests, not treating patients’ pain, and not communicating important handoffs.^6^ Additionally, burnout has been associated with substance use, relationship issues, depression, and suicide.^5^,^7^,^8^

Originally, burnout was believed to manifest in those who practiced medicine for a prolonged amount of time. However, recent evidence has shown that burnout may begin as early as medical school and residency.^9^–^10^ In fact, recent studies on emergency medicine (EM) residents reported burnout rates ranging from 65–76%.^9^,^11^ Across all fields of medicine, residents have shown higher rates of burnout when compared to medical students and early-career physicians.^9^,^10^

In response to this data indicating early onset of burnout, the Accreditation Council for Graduate Medical Education (ACGME) has pushed for initiatives on resident wellness, revising their Common Program Requirements for accredited residencies and fellowships to “emphasize that psychological, emotional, and physical well-being are critical in the development of the competent, caring, and resilient physician.”^12^ A needs-assessment performed on EM residents has shown that residents believe the topic of wellness is relevant and valuable to their career. However, they do not feel comfortable with their knowledge of wellness principles.^13^ As medical educators and residency program leaders work to meet the ACGME requirements and emphasize resident well-being at their institutions, they will benefit from current evidence on the various assessment tools, individual- and organizational-level interventions. This article provides a narrative summary of the literature and recommendations for best practices for assessing burnout and creating wellness initiatives in graduate medical education (GME), focusing on EM residency programs.

## CRITICAL APPRAISAL OF THE LITERATURE

This is the third in a series of evidence-based best practice reviews from the Council of Residency Directors in Emergency Medicine (CORD) Best Practices Subcommittee.^11^,^12^ Two authors independently performed a search of PubMed for articles published from inception to April 26, 2018, using a combination of the following search terms: wellness, wellness programs, well-being, stress, burnout, physicians, residents, and health personnel. Articles were prioritized if they focused on EM residents. When EM-specific literature was not available, we included relevant articles pertaining to wellness among other healthcare personnel. Bibliographies of all relevant articles were reviewed for additional studies. The literature search yielded 2931 articles, which were screened by two authors to include any papers addressing the following themes: assessment tools for wellness/burnout; individual interventions to treat/prevent burnout; and organizational interventions to treat/prevent burnout. After screening, 112 articles were deemed directly relevant for inclusion.

We provide level and grade of evidence for each statement according to the Oxford Centre for Evidence-Based Medicine criteria (Tables 1 and 2). When supporting data were not available, recommendations were made based upon the authors’ combined experience and expert opinions. Prior to submission, the manuscript was reviewed by the CORD Best Practices Subcommittee for additional comments and identification of missed references. It was additionally posted to the CORD website for two weeks for review from the CORD community.

## DISCUSSION

### Assessment of Burnout/Wellness

The first step in any successful intervention is a needs assessment. While large population-level studies have demonstrated a need to improve physician well-being,^2^ a targeted needs assessment and problem identification is suggested prior to any specific intervention, according to the second step in Kern’s six-step approach to curriculum development.^17^ A comprehensive needs assessment first requires identification of the population of interest (e.g., EM residents in a single training program) and the specific problem (e.g., burnout). A common pitfall is failure to narrow the scope of the problem that an intervention is designed to address. This can be challenging as there is lack of an agreed-upon definition for “burnout” or “wellness.”^18^,^19^ Because burnout is not listed in the *Diagnostic and Statistical Manual of Mental Disorders*-V,^20^ it is common for the term “burnout” to be incorrectly used to refer to anything ranging from depressive symptoms to increased work demands. For this reason, surveys that ask participants to self-identify their perceived burnout as a single-item Likert response (e.g., “rate your level of burnout”) are not valid measures.

Wellness may be variably defined as “work-life balance,” “life satisfaction,” or “the absence of burnout,” depending upon the context of an intervention.^21^ It is not sufficient to state that an intervention is designed to “promote wellness” without first specifying the framework that defines wellness in a particular circumstance. Knowledge of the operating characteristics of commonly used assessment tools can facilitate problem identification. For example, burnout,^22^ depression,^23^ anxiety,^24^ perceived stress,^25^ and resilience,^26^ each have validated assessment tools for measurement in different demographic groups, such as medical students, physicians, and the health professions in general. Table 3 provides a review of the key tools available to assess these components, including whether it was validated specifically in physician populations.

Other things to consider when selecting an appropriate assessment tool include cost, ease of completion, potential confounding factors, and whether it has been studied in target populations. Cost may be a significant factor for selection of the assessment tool. Although some surveys such as the Maslach Burnout Inventory (MBI-HSS) require payment for use,^27^ many tools are available at no cost. Survey length can have a negative impact on the response rate, influencing the tool selected. One must also consider the influence of confounding factors; for example, the MBI-HSS emphasizes the assessment of thoughts and feelings in the workplace and is less likely to be confounded by non-workplace related factors than other tools.^28^ Lastly, not all tools have been created with attending or resident physicians in mind. This particular population may face unique stressors and challenges and may not exhibit the same response patterns as the general public.

The MBI-HSS is probably the most widely recognized measure of burnout in physicians. Maslach and colleagues operationally defined burnout for workers in the helping professions (e.g., healthcare workers, first responders, social workers) as a combination of three domains: EE, DP and lack of PA.^22^ The original authors preferred that burnout be reported as a continuous variable (i.e., high, medium, or low) rather than as a dichotomous one (i.e., burnout or no burnout).^22^ Since then, multiple researchers have applied their own criteria for burnout using the three subscales, which has resulted in at least 47 distinctly different definitions of burnout using the MBI-HSS alone.^29^ Therefore, when considering the assessment of burnout, it is essential to remember the framework used and the test characteristics of the tool selected.

Ongoing assessment and program evaluation are essential to determine the efficacy and successful achievement of goals and objectives. The interval of measurement can vary from weeks to months depending upon the scope and outcome of interest. For example, optimism is considered a relatively stable quality^30^ and unlikely to be sufficiently changed by a single four-week curricular intervention; therefore, an optimism/pessimism assessment tool (e.g., the Revised Life-Orientation Test) would be ill-suited for a pre-/post-measurement in this context. It is also important to note that survey fatigue can result in lower quality data by introducing bias, including non-response bias.^31^

Finally, a distinction should be made between institution- and individual-level assessment. The updated ACGME Common Program Requirements emphasize the need for mechanisms to identify residents at risk of burnout, depression, substance abuse, suicidal ideation, and the potential for violence.^32^ They specify the need for program-level assessment, individual-level assessment, and self-screening measures. The ACGME has endorsed the MBI-HSS, Mayo Clinic Well-Being Index, Patient Health Questionnaire-9, and the Professional Quality of Life Scale as potential tools for assessment at the program-level and individual-level.^12^

While each of the recommendations and tools discussed above can be used for assessment of either the program or individual residents, extra caution should be taken when assessing specific residents who may be at risk. Evidence exists that trainees at risk of suicide completion are often difficult to identity,^33^,^34^ and overreliance on any one assessment tool should be avoided. Identification of at-risk residents should be a composite evaluation of attention to professional duties, clinical performance, known work-related or personal crises, and concerning changes in behavior or language used by the resident.^35^

### Individual Interventions

Over the last two decades, there have been increasing calls for high-quality randomized controlled trials of specific burnout interventions. A recent, systematic review summarizing psychosocial interventions for managing physician workplace stress identified over 15,000 studies containing the keywords physician, stress, and burnout. Unfortunately, only 20 were intervention studies, and among these only 12 included pre- and post-intervention assessments.^36^ None were deemed high-quality or specific to EM residents.^36^ A similar systematic review in GME found only three intervention studies.^21^ Several barriers common to medical education research exist, including variable definitions, small sample sizes, the ethics of randomization, difficulty with individual assessments, long-term follow-up, and external validity. Among the higher-quality intervention studies, relaxation training, behavioral interventions, and self-care were demonstrated to be most effective.^21^,^36^

**Table:** Best Practice Recommendations for Assessment

Clearly define the purpose and need for an intervention, then choose an assessment tool for gathering data, establishing a baseline, measuring outcomes, and/or monitoring. (Level 5, Grade D) Understand that there is variability in the definitions of burnout, wellness, and other outcomes. Be clear on the limitations of any defining criteria being used when interpreting the results of any assessment tool. (Level 3a, Grade C) Each assessment tool has unique benefits and limitations. Consider the intended purpose, cost, length, confounding factors, and population of interest when selecting a tool. (Level 3b, Grade C)

Mindfulness is defined as an awareness of the present situation while accepting thoughts, emotions, and physical sensations.^37^ Being mindful can be a valuable tool for combating burnout. One randomized controlled trial focusing on mindfulness-based coping strategies demonstrated enhanced well-being and decreased rates of burnout after the intervention.^38^ Another study using a modified mindfulness-based stress reduction program, consisting of a workshop followed by eight weeks of daily meditation, showed improvement in general health and stress.^39^ Similar mindfulness and meditation studies have been reported in the undergraduate and the nursing wellness literature.^40^–^44^ More feasible interventions, such as 10–20 minute mindfulness meditation for 30 days and a one-hour online module focusing on “mind-body skills training,” have also been demonstrated to reduce stress and burnout.^45^–^52^ Mindfulness and self-awareness can be enhanced by journaling, narrative medicine, and reflective questioning, potentially reducing burnout rates,^50^–^51^ although other studies have not shown improvement in burnout.^52^

Focused training in behavioral skills, such as cognitive reframing, self-compassion, and empathy, can also improve wellness.^56^–^61^ A survey of winners of the American Medical Association Foundation’s Pride in Professions Award identified self-compassion and self-care as key components for combating burnout.^62^ A study of internal medicine residents found that residents who employed strategies of active coping and positive reframing of difficult situations had lower rates of EE and DP.^25^ Similar results have been found in the nursing literature.^63^ While behavioral skills training has been shown to improve physician wellness, stress management training has not been shown to improve burnout rates in physicians.^52^,^64^

Physicians who are able to engage in regular self-care, such as ensuring adequate physical health, sleep, nutrition, and exercise, consistently have lower rates of burnout.^65^–^70^ Although scheduling can be a challenge, regular exercise improves physician wellness.^71^,^72^ Incentivized exercise programs, however, have not been shown to improve physician wellness. 52,64 A study of approximately 7000 surgeons showed that those who visited their primary care physician (PCP) in the prior 12 months had lower rates of burnout and a higher quality of life.^71^ Unfortunately, a recent survey reported that nearly half of surgical residents reported not being able to visit their PCP regularly and gaining weight during residency.^73^ Fortunately, the literature demonstrates that we can teach our trainees how to implement effective behavioral change plans to improve personal behaviors such as exercise, nutrition, sleep, personal hygiene, and emotional health.^74^

In addition to physical health, psychological health is also important. A survey of anesthetists and intensivists showed that alcohol dependence, abuse of sedative medications, and overeating were correlated with higher rates of depression and burnout.^75^ Another study looking at residents found that only 24% of residents who felt they needed mental health care sought treatment.^76^ Residents cited lack of time, concern of confidentiality, and cost as barriers to treatment. Encouragingly, studies looking at physicians who participated in either individual or group counseling with a trained professional showed a lasting reduction in EE for up to three years.^77^

Building a strong friend and colleague support network is associated with lower burnout scores.^78^–^81^ Indeed, Dr. Maslach herself recently proposed that civility and teamwork are fundamental to physician wellness.^79^ A survey of 198 physicians-in-training showed that loneliness was significantly associated with both personal and professional burnout.^81^ Peer- and faculty-mentoring programs may help create these needed support networks as they are correlated with lower burnout rates.^82^

For residents currently suffering from burnout, the road back to wellness begins with an empathetic discussion with a program faculty member or impartial third party (e.g., designated institutional officer). Confidential meetings need to ensure the resident’s health and safety and to develop a personalized wellness plan specific to the type of burnout, circumstantial or existential. Circumstantial burnout originates from acute, self-limited situations.^83^ Helpful interventions may include speaking with a professional therapist, developing strategies for mitigating life or workplace difficulties, creating daily time for self-care, and even providing brief time off from clinical duties.^83^ Existential burnout originates from a chronic loss of joy from the practice of medicine itself. Helpful interventions may include speaking with a professional therapist, examining the origins of burnout, developing better relationships with patients and colleagues, and even reshaping the resident’s professional identity.^83^ Consistent follow-up ensures that the resident is recovering and provides opportunities to identify when other interventions are needed.

It is important to note that while individual interventions to improve wellness show some promise, evidence suggests that intervention programs based on the individual are associated with only small benefits on burnout and should be supplemented by the adoption of organizational approaches.^84^

### Organization-Level Interventions

In addition to individual interventions, institutional, organizational and departmental wellness committees have been widely recommended as a strategy to address physician, staff, and trainee wellness.^40^,^85^,^86^ Committees should be composed of residents and faculty members, who meet regularly to assess, analyze, and develop systematic initiatives to improve the clinical learning environment for all.^40^

Committee members should contribute to the creation of a wellness program or curriculum. Lefebvre discussed the key components of a resident wellness program, which include creating a safe space; having one-on-one meetings with residents; and designing residency events focused on physical, mental, social, intellectual, and community wellness.^82^ Wellness programs should combine both passive (e.g., safe places, lectures, website resources) and active (e.g., meetings, workshops, outings, small group activities, service projects, gym access) strategies.^86^ Other components could include strategies to help deal with the wide range of issues that may be encountered during residency, such as stress management, behavioral issues, marriage or family problems, financial troubles, substance abuse, disruptive colleagues, or mental health issues.^85^,^87^–^88^

**Table:** Best Practice Recommendations for Individual Interventions

Mindfulness training should be incorporated into residency training to improve wellness and reduce burnout (Level 1b, Grade B). Consider incorporating behavioral interventions, such as reframing, self-compassion, and empathy into residency training (Level 4, Grade C) Encourage self-care with respect to physical, psychological, and emotional health. This should include an emphasis on sleep, healthy eating, regular exercise, development of social and professional support networks, PCP visits, resources for substance abuse, and counseling or mentoring programs (Level 4, Grade C) Program faculty should meet privately with residents potentially suffering from burnout to identify the unique causes and appropriate interventions. Close follow-up meetings should assess improvement (Level 4, Grade C)

*PCP*, primary care physician.

In addition to institutional programs, residency curricula have been shown to be beneficial.^45^,^86^, ^90^–^97^ Studies recommend a multicomponent wellness curriculum, including resilience, professionalism, emotional wellness, physician suicide, social wellness, financial wellness, team building, and mindfulness.^89^–^90^,^98^ Occupational wellness components have also been suggested, including ethical and interpersonal encounters (eg, difficult patients, difficult consultants).^45^,^89^,^94^ Many residency programs have been using Balint groups to supplement their resident wellness initiatives.^91^–^92^,^99^ Balint groups focus on the doctor–patient relationship by enhancing communication skills among physicians; however, studies show variable results on the ability of Balint groups to improve wellness.^52^,^64^,^87^,^88^,^95^,^100^ While curricula should be program-driven, most studies recommend the inclusion of out-of-hospital components, including retreats, workshops, and social outings.^86^,^89^,^93^

Studies of curricula that emphasize mindfulness, resilience training, and stress management have demonstrated improved physician wellness and reduction in burnout scores.^101^–^103^ While it seems intuitive that mindfulness and resilience, the ability of an individual to effectively cope with and adapt to adverse situations, would have positive effects on the wellness and burnout of EM residents, it is useful to review the literature on systemwide interventions in these areas. One particular study by Krasner, involving a longitudinal curriculum on mindful communication, noted both short-term and sustained improvements in well-being and attitudes associated with patient-centered care.^104^ Another study by West involved a nine-month curriculum, in which physicians met in small groups on a biweekly basis for discussion groups that incorporated elements of mindfulness, reflection, shared experience and small-group learning. This curriculum improved rates of high DP, which was sustained at 12 months, as well as improvements in empowerment and engagement at work.^101^

Evaluation of a stress management and resiliency training (SMART) curricula found improved stress, anxiety, and overall quality-of-life scores among both radiology and internal medicine faculty physician participants.^105^,^106^ Another institutionally implemented resiliency curriculum for palliative care and neonatal providers led to improved compassion sensitivity and burnout scores after completion of the program.^107^ Critical care fellow participants in a SMART program intervention felt the training provided them with tools to apply during stressful situations, but did not demonstrate improved burnout scores.^108^ Similarly, Maher found that a departmentally-instituted educational program designed to improve surgical resident performance during stressful scenarios showed a trend toward improved performance scoring but no difference in anxiety levels. However, 91% of residents rated the stress training as valuable.^108^–^109^ While residents and fellows consistently report subjective benefit from resiliency training, improvement in burnout scores have not been reliably demonstrated. Resiliency training is not the only intervention that has failed to show an improvement in burnout scores. Studies of stress management workshops and training sessions have also demonstrated no difference in physician burnout rates.^52^,^64^ Similarly, a recent study evaluated burnout scores of EM residents before and after implementation of a corporate wellness intervention, “The Happiness Practice.” The resident burnout scores did not improve; in fact, 43% of residents stated that this intervention worsened their overall level of burnout.^110^

Despite lack of overwhelming evidence that resilience training programs improve burnout scores in residents, there are several studies that demonstrate the importance of the personal trait of resilience in preventing burnout.^99^,^101^,^111^–^112^ One study assessed the role of resilience in the relationship between burnout and health among critical care professionals and found that resilience was a key component in mitigating burnout syndrome.^113^ Another study demonstrated that a resilience-building intervention for physicians improved meaning and work engagement while also reducing DP, with sustained results at 12 months.^101^ A well-diversified pool of social resources and interests, together with realistic expectations and good self-knowledge, were found to support sustainable coping in a study of 200 physicians from multiple specialties and career stages.^111^ A 2015 survey of 616 ED healthcare professionals demonstrated that an individual’s coping style may be a predictor of burnout and compassion fatigue.^112^ This study found that task-oriented coping is associated with a decreased risk of burnout in contrast to emotion and avoidance coping styles.^112^ The cumulative data strongly suggests that resilience is a burnout mitigating factor; therefore, residency programs should consider making resilience training programs available to residents either on an individual or systemwide level.

Resident wellness can also be optimized by evaluating workplace and workflow interventions.^114^–^116^ Workflow interventions include electronic health records (EHR) optimization, improving staff-provider communications, and offloading both clinical and non-clinical tasks that could be performed by other members of the medical team.^116^–^117^ EHR efficiency training and the use of scribes or dictation devices has been shown to decrease stress and burnout in attending physicians.^114^–^115^ Additionally, delegating administrative tasks to non-clinical staff has been shown to improve overall wellness.^115^,^118^ Improved workplace conditions, including optimizing workflow, can lead to overall decreased resident physician stress and burnout.

Resident schedules are often a topic of discussion in the medical education community. Since the ACGME duty-hour changes over the past decade, many studies have evaluated the effect of duty-hour restrictions on patient outcomes and resident wellness across specialties.^64^,^117^,^119^–^121^ Studies suggest that working >80 hours per week correlates with higher rates of burnout when compared to working <80 hours per week.^120^ Another study found that working >60 hours per week was associated with higher rates of burnout and psychological morbidity.^121^ According to multiple meta-analyses, the implementation of the ACGME guidelines for duty hours resulted in an increase in resident wellness and PA, as well as a decrease in EE, DP, and burnout.^64^,^117^,^119^ Additionally, it has been suggested that implementing protected sleep time, an uninterrupted period of sleep during overnight call, better aligns with circadian physiology and can improve fatigue and prevent burnout.^117^ This may be of particular importance during off-service rotations and transitions between rotations.

Scheduling can also affect the ability to access personal medical care. Resident physicians are significantly less likely to have a PCP than their demographically-similar peers outside of medicine.^122^ According to a study by Cedfeldt and colleagues, residents in a department with a personal time policy were more likely to find time to fulfil personal needs.^122^ Residents who took personal time off had significantly higher proportions of positive experiences and emotions, lower proportions of negative experiences and emotions, higher satisfaction with their career choice, and less perceived stress.^123^ Another recent study looked at implementation of a universal well-being assessment for residents, by scheduling each resident for a mental health evaluation based on the residents’ schedule with the ability to opt out.^124^ The study found that 93% of residents participated in the program, increasing resident utilization of mental health resources.^124^ The residents also felt that the scheduling provided convenience, allowing residents to prioritize their mental health and self-care.^124^

In addition to total hours worked, many residents also reported that scheduling directly affects their wellness.^122^,^125^ Lack of control over their schedule can make it challenging to find time with family and friends, increasing burnout.^125^ EM residents have been shown to appreciate shift work guidelines that focus on the importance of circadian scheduling (i.e., advancing shift times progressively from day to evening to night) but, if given one option, prefer having the ability to request days off and have a full weekend off.^126^ A recent study showed that 93% of EM residency programs allow residents to make schedule requests.^127^ Programs should consider having “special requests” days each month to allow residents to attend important life events and to ensure residents have protected time off to attend healthcare appointments. Program leadership should work with residents to identify ways to balance increased resident control of scheduling while ensuring appropriate emergency department coverage. Program leadership should engage residents in the re-evaluation of current scheduling/staffing models, especially night shift models. Residency programs should recognize that giving residents more direct control of their schedule, schedule requests and sleeping patterns may help improve overall wellness.

## LIMITATIONS

It is important to consider several limitations with respect to this article. While we used multiple methods to identify relevant articles, it is possible that some articles may not have been identified by the current review. However, we used an inclusive search strategy, as well as review of the bibliographies of included articles to identify the most relevant literature. We also included several nationally recognized experts on wellness and engaged in pre-publication peer review by the CORD Best Practices subcommittee and the larger CORD community.

Additionally, article selection was based upon relevance to the specific themes selected. The topic of wellness is extensive, and we selected for review only specific components deemed to be most relevant to the clinician educator. Finally, while preference was given to data directly evaluating wellness assessment and interventions in EM residency programs, the data were limited. Therefore, when data specific to EM residency programs were not available, we used data from other medical residencies and fields as a surrogate.

## CONCLUSION

This paper provides an evidence-based review of the literature on wellness in residency education. Strategies for identification, as well as individual and system-level interventions that have shown improvement in resident wellness are discussed along with recommendations for best practices. After reading this paper, readers should have a greater understanding of how to engage in wellness assessment and intervention at their home institution.

**Table:** Best Practice Recommendations for Institutional Interventions

Creation of a departmental or institutional wellness committee is vital, should include residents and faculty, and should be involved in the planning and creation of wellness interventions, including curricular design (Level 5, Grade D) Institutional resources should be dedicated toward resident wellness programs (Level 3b, Grade C) Wellness curricula should address multiple domains of wellness and contain both passive and active components (Level 3b, Grade C) Consider developing workflow interventions, such as EHR optimization, and providing increased administrative support (Level 3b, Grade C) Schedules should be optimized to allow residents to request personal time off, to avoid excessive work hours, and to ensure appropriate transitions and circadian rhythms (Level 2b, Grade C)

*EHR*, electronic health records.

**Table:** CORD Best Practice Committee 2018–19

Michael Gottlieb, MD- Co-Chair Rush University Medical Center
John Bailitz, MD- Co-Chair Northwestern University, Feinberg School of Medicine
Richard Byyny, MD Denver Health Medical Center
Michelle Clinton, MD Virginia Tech/Carilion Medical Center
Benjamin Cooper, MD University of Texas- Houston
Molly Estes, MD Loma Linda University
Puja Gopal, MD University of Illinois in Chicago
Jaime Jordan, MD University of California- Los Angeles
Albert Kim, MD Washington University in Saint Louis
Andrew King, MD The Ohio State University
Sreeja Natesan, MD Duke University Medical Center
Melissa Parsons, MD University of Florida- Jacksonville
Greg Tudor, MD University of Illinois- Peoria
Brian Wood, MD Yale University Medical Center

## Figures and Tables

**Table 1:** Oxford Centre for Evidence-Based Medicine criteria.^16^

Level of evidence	Definition
1a	Systematic review of homogenous RCTs
1b	Individual RCT
2a	Systematic review of homogenous cohort studies
2b	Individual cohort study or a low-quality RCT*
3a	Systematic review of homogenous case-control studies
3b	Individual case-control study**
4	Case series or low-quality cohort or case-control study***
5	Expert opinion

* defined as <80% follow up;

** includes survey studies;

*** defined as studies without clearly defined study groups.

*RCT*, randomized controlled trial.

**Table 2:** Oxford Centre for Evidence-Based Medicine grades of recommendation.^16^

Grade of evidence	Definition
A	Consistent level 1 studies
B	Consistent level 2 or 3 studies or extrapolations* from level 1 studies
C	Level 4 studies or extrapolations* from level 2 or 3 studies
D	Level 5 evidence or troublingly inconsistent or inconclusive studies of any level

* “Extrapolations” are where data were used in a situation that has potentially clinically important differences than the original study situation.

**Table 3:** Selected assessment tools.

Name of instrument	Brief description and cost	Access	Pros	Cons
Inventory - Human Services Survey (MBI-HSS)	22 items 10 minutes to complete $15 per individual report; $50 for the manual; $250 add-on to analyze group results	http://www.mindgarden.com/117-Maslach-Burnout-Inventory	Most widely used and recognized Validated in physician populations	Significant cost Variable methods of interpretation of results
Copenhagen Burnout Inventory (CBI)	19 items 10 minutes to complete Free	http://www.arbejdsmiljoforskning.dk/upload/cbi-scales.pdf	Assesses burnout in the context of work-related and patient-related factors	Less commonly used tool than the MBI for assessing burnout
				
Professional Quality of Life Scale (ProQol)	30 items 15 minutes to complete Free, but must credit the author	http://www.proqol.org/Home_Page.php	Validated in multiple populations and has demonstrated good reliability Assesses compassion and satisfaction, burnout, and secondary traumatic stress	One of the longer assessment tools. Recommended to complete in entirety rather than divide by subscales
Mayo Clinic Well-Being Index (WBI)	9 items < 5 minutes to complete Free for individuals; $10K license plus $5K yearly for organizations	https://www.mededwebs.com/physician-well-being-index	Validated in physicians Provides self-directed learning resources	Significant cost More useful as a screening tool than a detailed assessment
WHO Well-Being Index (WHO-5)	5 items < 5 minutes to complete Free	https://www.psykiatri-regionh.dk/who-5/Pages/default.aspx	Widely validated in multiple populations Can be used to monitor changes in well-being	Not well studied in physicians Does not specify any work-related factors
Perceived Stress Scale (PSS)	14 items 10–15 minutes to complete Free	http://www.psy.cmu.edu/~scohen/scales.html	Can be used to monitor changes in perceived stress	Not validated in physicians
Patient Health Questionnaire (PHQ-2)	2 items < 2 minutes Free	http://www.phqscreeners.com/	Widely used and recognized	Some concerns about reliability given brevity of test
Connor Davidson Resilience Scale (CD-RISC)	25 items 10 minutes to complete Cost dependent on agreement with authors	http://www.connordavidson-resiliencescale.com/index.php	Well validated in the general population	Not well studied in physicians Resilience may be a more enduring trait and less subject to change based on a single intervention
Single-item measures of emotional exhaustion (EE) and depersonalization (DP)	2 items derived from the full MBI-HSS < 2 minutes to complete Free	West CP, Dyrbye LN, Sloane JA, Shanafelt TD. Single-item measures of EE and DP are useful for assessing burnout in medical professionals. J Gen Intern Med. 2009;24(12):1318–21.	Validated in physician populations	Some concerns about reliability given brevity of test
